# Pediatric Crohn’s Disease With Avoidant and Restrictive Food Intake Disorder (ARFID) Resulting in Failure to Thrive: A Case Report

**DOI:** 10.7759/cureus.69972

**Published:** 2024-09-23

**Authors:** Vladimir Valencia, Michael J Cosare, Maristela Soberano, Lauren M Toledo, Madhura Butala

**Affiliations:** 1 Research, Lake Erie College of Osteopathic Medicine, Bradenton, USA; 2 Pediatrics, Ascension St. Vincent's Riverside, Jacksonville, USA

**Keywords:** arfid, avoidant-restrictive food intake disorder, failure-to-thrive, inflammatory bowel disease, pediatric crohn's disease

## Abstract

Failure to thrive (FTT) refers to a condition where a child does not gain weight or grow at the expected rate for their age and gender. An accepted definition includes a weight less than the lowest acceptable range on standardized growth charts. FTT is often a diagnostic challenge for providers treating children with mixed etiologies. This report discusses the case of an 11-year-old female with a diagnosis of Crohn’s disease and avoidant and restrictive food intake disorder (ARFID). Management by an interprofessional healthcare team has been difficult, given the multifactorial nature of the patient’s weight loss. This report suggests that behavioral and psychological aspects, such as aversion to eating and reluctance to experiment with different foods, may align with symptoms of Crohn's disease in children. It also emphasizes the importance of timely diagnosis and appropriate interventions, as they can mitigate psychological and developmental setbacks.

## Introduction

Failure to thrive (FTT) refers to a condition where a child does not gain weight or grow at the expected rate for their age and gender. The precise definition continues to be debated; however, an accepted definition includes a weight less than the fifth percentile on standardized growth charts, less than the 80th percent of median weight for height/length ratio, or a decrease in weight percentile of more than two major percentile lines on the growth chart [1]. FTT poses a significant risk factor for malnutrition, which, if left untreated, can lead to significant comorbidities including developmental delay, stunted growth, and other long-term sequelae [2]. Multiple studies investigating the effects of poor nutrition on cognitive development have shown an association of FTT with decreased IQ, behavioral challenges, impaired communication skills, and multiple learning disabilities [1]. It is therefore essential that the diagnosis and appropriate management of FTT be accurate to best mitigate these long-term sequelae. However, FTT remains a diagnostic challenge for providers treating children with mixed etiologies.

An organic cause of FTT is Crohn’s disease. Crohn’s disease is one of the two main types of inflammatory bowel diseases (IBDs) that often affects the small intestine as well as the proximal colon; however, it can also affect other portions of the gastrointestinal tract. The clinical presentation and course of the disease often exhibit many of the same characteristics as those in older patients; however, the risk of malnutrition is more severe in the developing child. Most children are diagnosed in the early teen years, but subgroups with very early onset and infantile Crohn’s are diagnosed much earlier and follow a unique course [3].

IBD has been noted to have a positive correlation with avoidant and restrictive food intake disorder (ARFID). ARFID is characterized by an avoidance or restriction of food intake, often due to fear of adverse consequences such as choking or vomiting, sensory sensitivity, or lack of interest in eating. ARFID differs from other eating disorders, such as anorexia nervosa or bulimia nervosa, as it is not primarily driven by body image concerns or the desire to lose weight. Rather, it is more due to an aversion to eating situations or certain foods. Certain studies have shown that individuals with active IBD and concomitant ARFID are significantly more likely to be at risk for malnutrition [4].

## Case presentation

The patient is an 11-year-old female who was initially seen in the office at the age of 10 due to concerns from her parents about hiccups, gastroenteritis, and mouth odor. She lived in India until the age of eight before moving to the United States.

Her parents report that she was always a picky eater as a child; however, over the prior six months, she would only eat a few bites of even her favorite foods and would refuse the rest. She described a sensation of fullness and an inability to finish her meals. At the time, she did not have any heartburn; however, she would feel a fullness in the back of her throat after eating. She had a history of constipation in the past requiring Miralax; however, her stools had been normal, and she was without abdominal pain. The episodes of feeding were now associated with the development of anxiety related to food. She would worry about mealtimes as soon as she woke up in the morning. Additionally, she would have episodes of hiccups once or twice per month, with episodes lasting 30 minutes. She was started on a trial of pantoprazole and underwent further workup.

**Figure 1:**
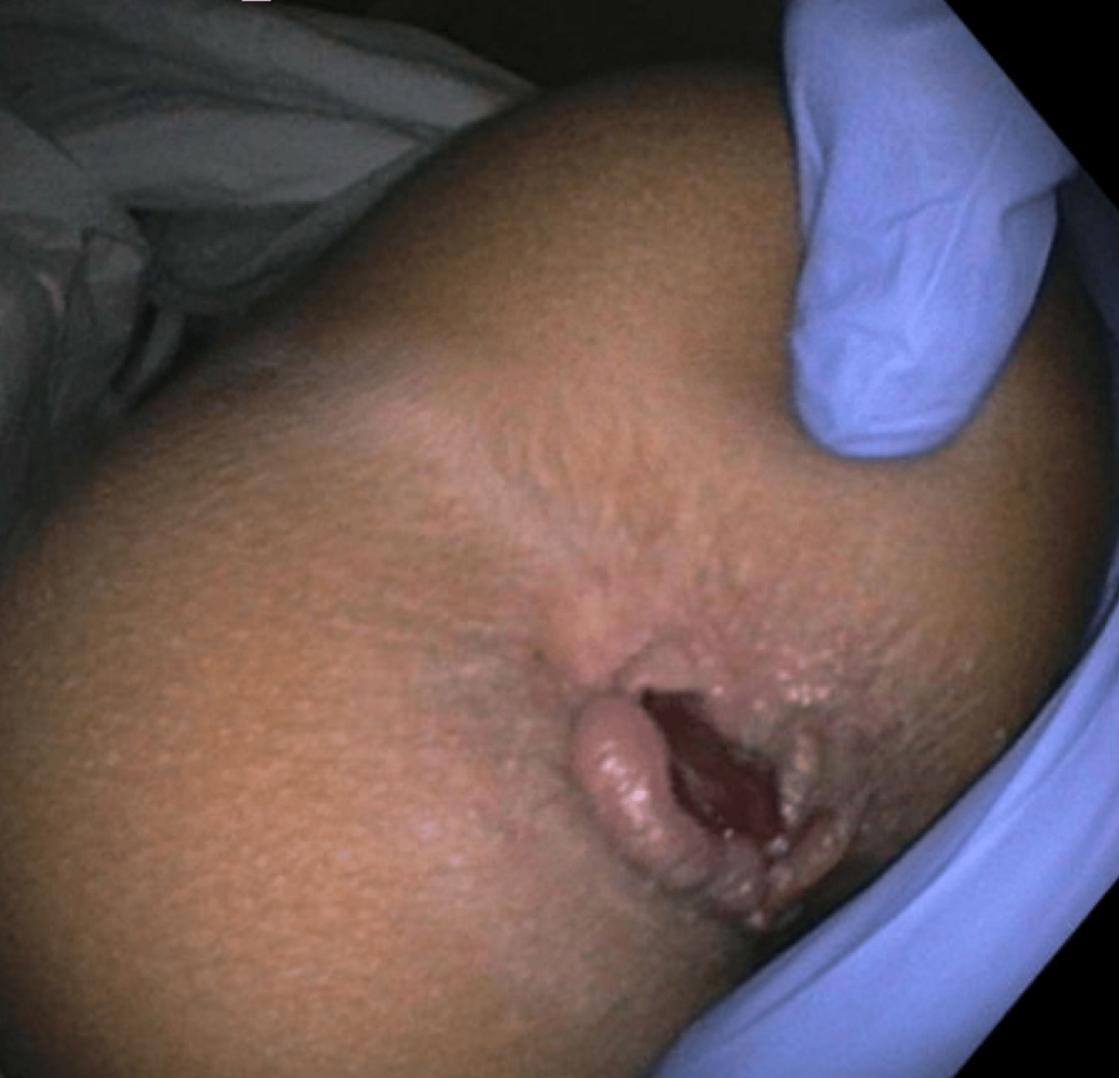
Visualization of external hemorrhoids and perianal skin tags

At her follow-up visit, she had new concerns about hard stools, along with blood in the stool that had been ongoing for one week. She was experiencing painful defecation and also noted skin-colored lesions protruding from the anal opening, for which she tried an over-the-counter hemorrhoid cream. Physical examination demonstrated evidence of hemorrhoids, along with perianal skin tags (Figure 1).

**Figure 2:**
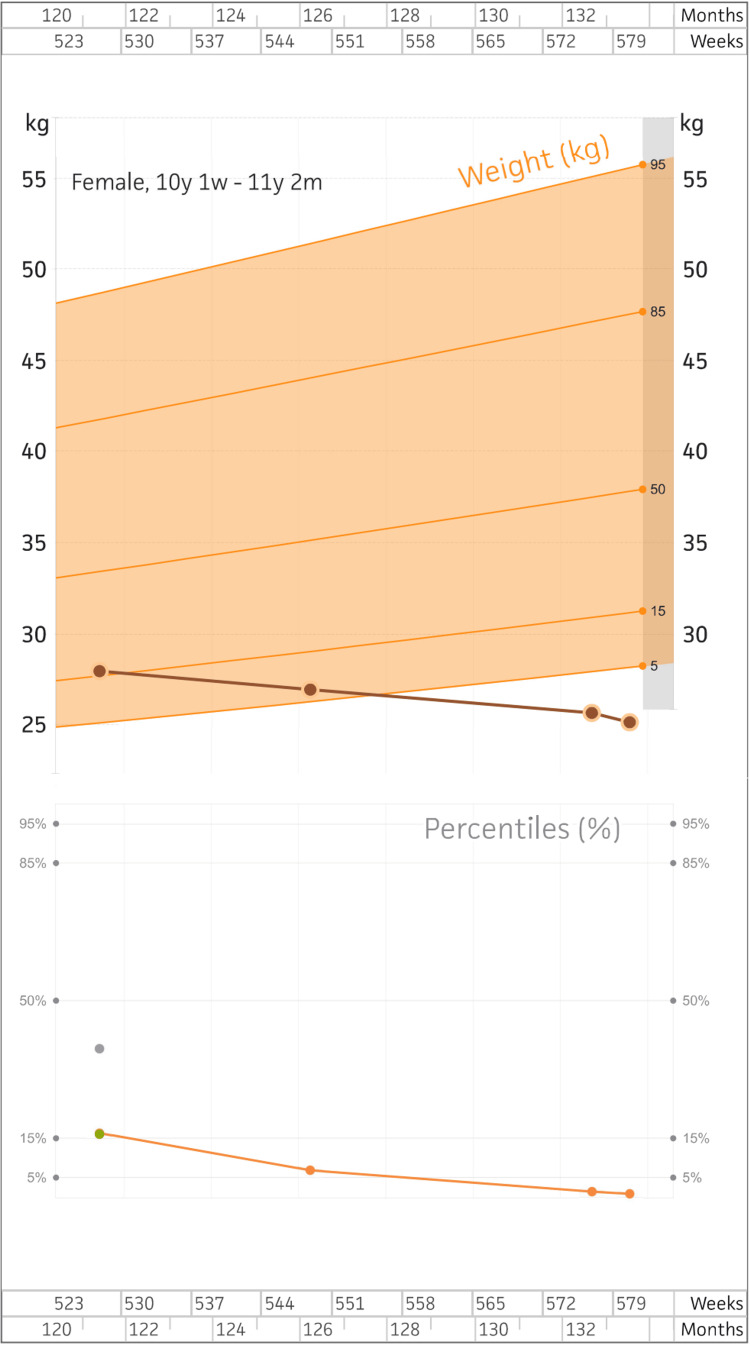
Patient growth chart trends from ages 10- to 11-years-old Stature, weight, and BMI values taken in-office over the course of one year demonstrate failure to thrive

She continued to have a poor appetite with an aversion to certain foods. She had refused to undergo the recommended lab work, as well as the psychiatry appointment. She reported that she had been too tired to attend school and that they had made arrangements for temporary home schooling. She had a recent gastroenterology appointment and was prescribed cyproheptadine, with plans to consider endoscopy and colonoscopy after the lab work was completed. She attempted feeding therapy, which she was unable to complete, and has since developed anxiety symptoms surrounding the idea of therapy; thus, those sessions were temporarily discontinued. At this point, her weight had dropped to 25.11 kilograms (55.36 pounds), as shown in Figure 2.

She underwent further extensive evaluation at a children’s hospital. Lab work (Table 1) was significant for a high erythrocyte sedimentation rate (ESR), C-reactive protein (CRP), and calprotectin. It also revealed low levels of albumin, vitamin D, serum iron, and iron saturation.

**Table 1:** Laboratory values

Lab Component	Value	Reference Range
Miscellaneous		
Albumin	3.4 g/dL	4.2-5.0 g/dL
Erythrocyte Sedimentation Rate	64 mm/hr	0-32 mm/hr
C-Reactive Protein	38 mg/L	0-9 mg/L
Calprotectin	1400 mcg/g	<50.0 mcg/g
Vitamin B12	362 pg/mL	232-1245 pg/mL
Vitamin D, 25-Hydroxy	28.4 ng/mL	30.0-100.0 ng/mL
Total Iron Binding Capacity	527 μg/dL	250-450 μg/dL
Unsaturated Iron-Binding Capacity	482 μg/dL	131-425 μg/dL
Iron	45 μg/dL	28-147 μg/dL
Iron Saturation	9%	15-55%
Stool Occult Blood	Positive	Negative
Complete Blood Count		
White Blood Cells	7.5 x 10³/μL	3.7-10.5 x 10³/μL
Red Blood Cells	4.73 x 10⁶/μL	3.91-5.45 x 10⁶/μL
Hemoglobin	12.5 g/dL	11.7-15.7 g/dL
Hematocrit	38.4%	34.8-45.8%
Mean Corpuscular Volume (MCV)	81.2 fL	77-91 fL
Mean Corpuscular Hemoglobin (MCHC)	32.6 g/dL	31.7-36.0 g/dL
Red Cell Distribution Width (RDW)	18.4%	11.7-15.4%
Platelet Count	295 x 10⁹/L	150-475 x 10⁹/L
Comprehensive Metabolic Panel		
Sodium	137 mmol/L	134-144 mmol/L
Potassium	3.4 mmol/L	3.5-5.2 mmol/L
Chloride	102 mmol/L	96-106 mmol/L
Blood Urea Nitrogen	5 mg/dL	5-18 mg/dL
Creatinine	0.56 mg/dL	0.42-0.75 mg/dL
Aspartate Aminotransferase (AST)	18 IU/L	0-40 IU/L
Alanine Aminotransferase (ALT)	12 IU/L	0-28 IU/L
Alkaline Phosphatase	112 IU/L	150-409 IU/L
Bilirubin	0.3 mg/dL	0.0-1.2 mg/dL
Gamma-Glutamyl Transferase (GGT)	18 IU/L	5-27 IU/L
Glucose	46 mg/dL	70-99 mg/dL

**Figure 3:**
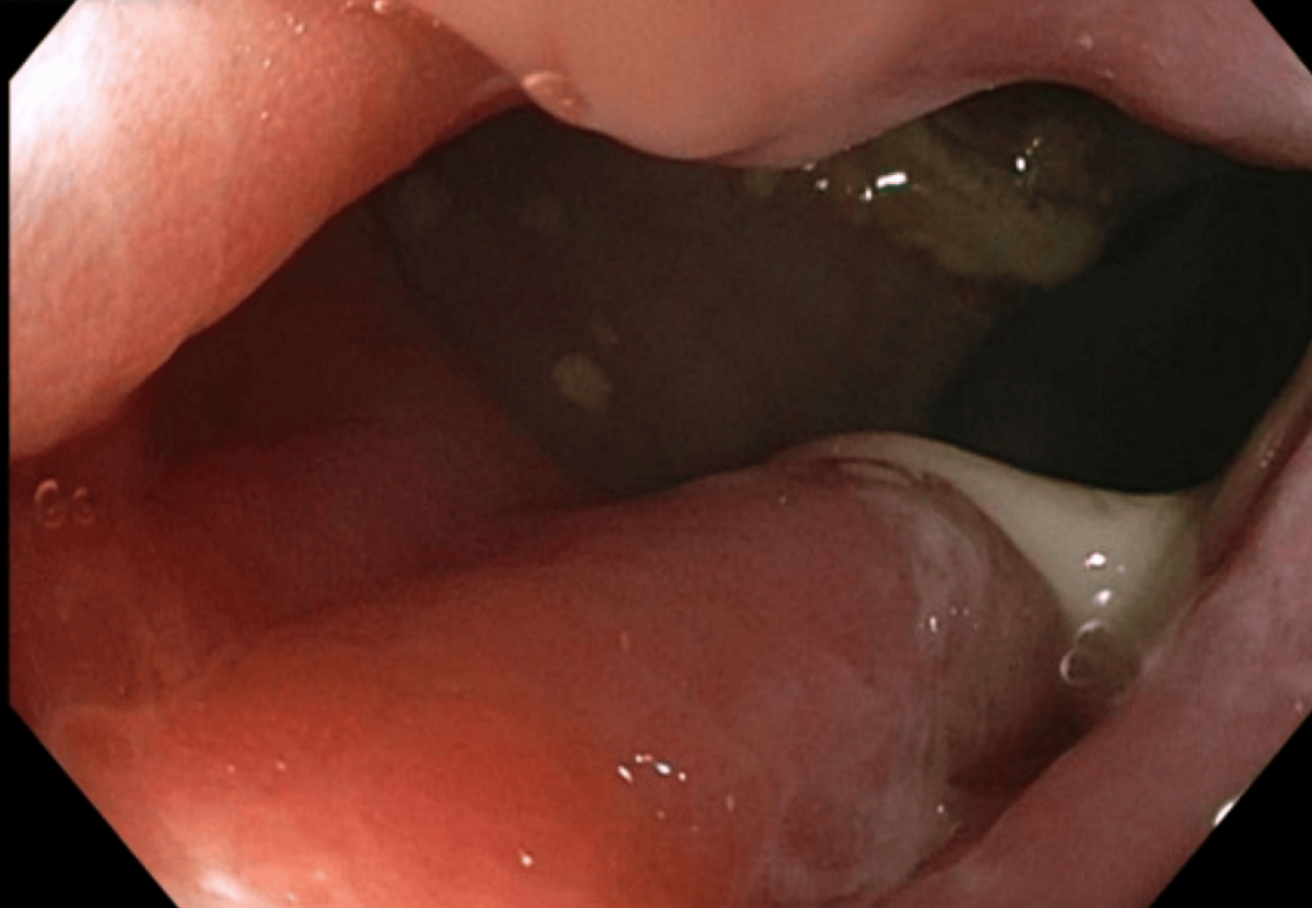
Rectum with diffuse area of moderately erythematous mucosa

She underwent an esophagogastroduodenoscopy, which was overall unremarkable. Colonoscopy findings demonstrated multiple perianal skin tags and a diffuse area of moderately erythematous mucosa in the rectum (Figure 3).

**Figure 4:**
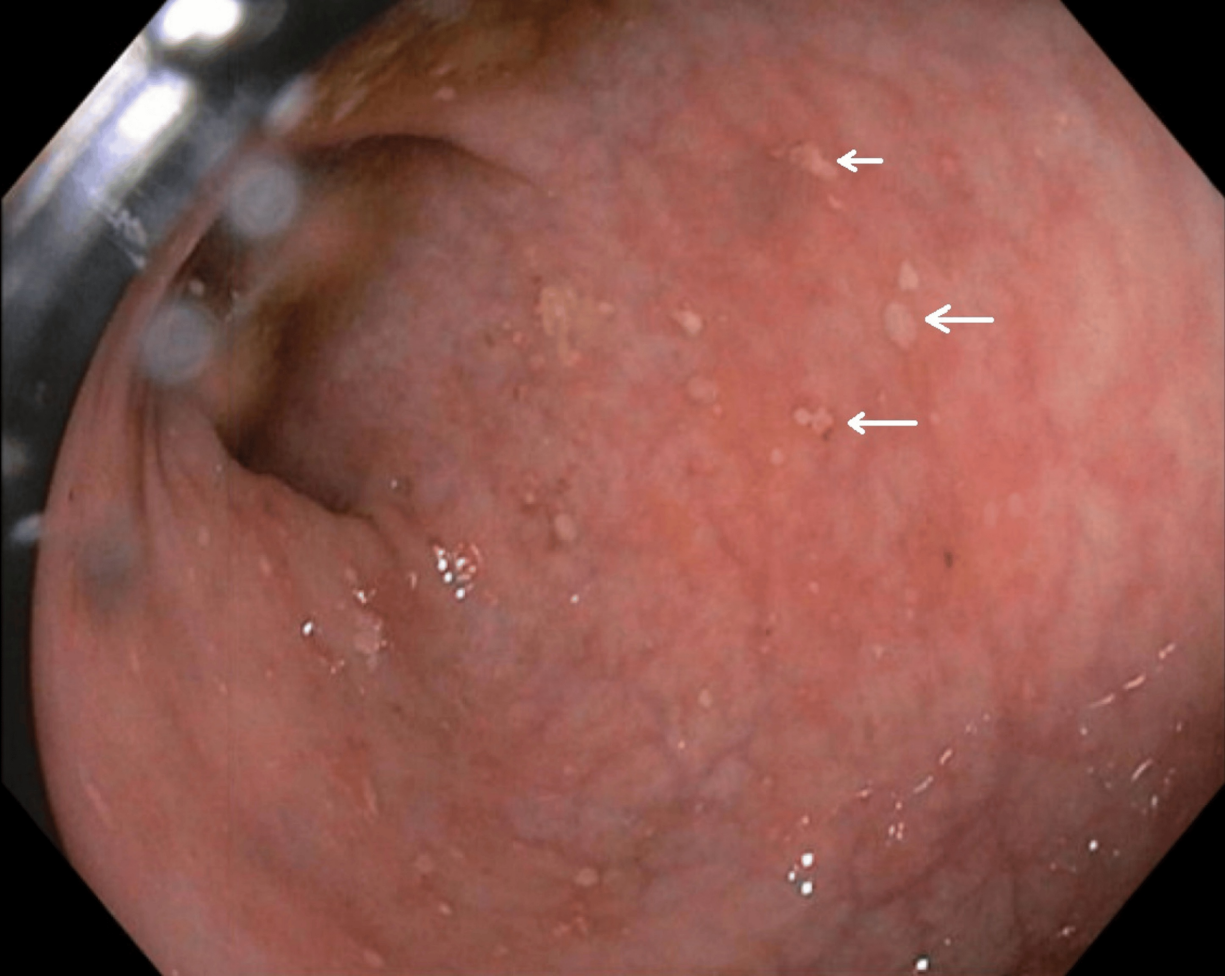
Aphthous ulcerations in mucosa (arrows)

A scattered area of shallow aphthous ulcerations in the mucosa was found from the rectum to the transverse colon (Figure 4).

**Figure 5:**
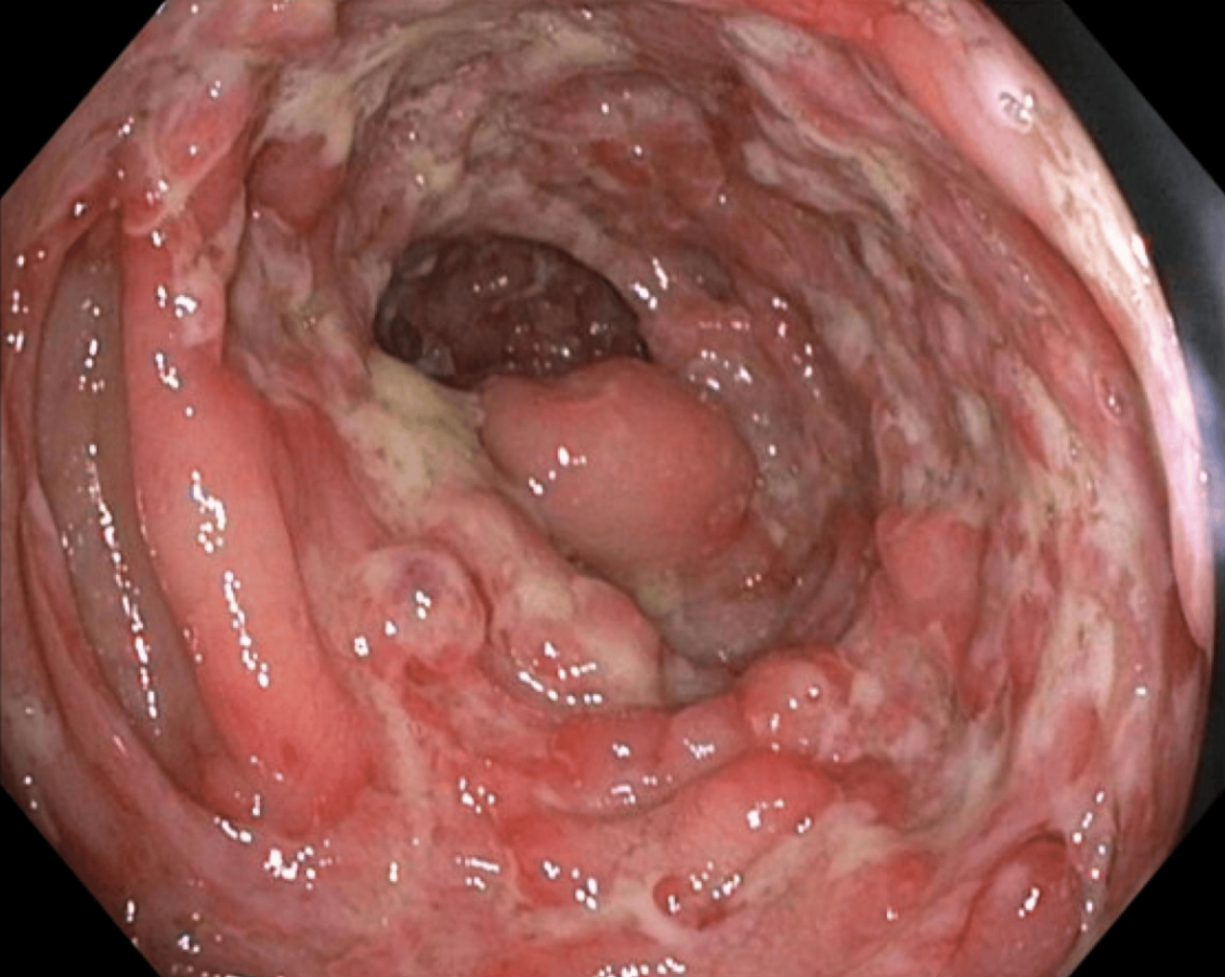
Ascending colon with severely erythematous, eroded, friable, inflamed, nodular, and pseudopolypoid mucosa

**Figure 6:**
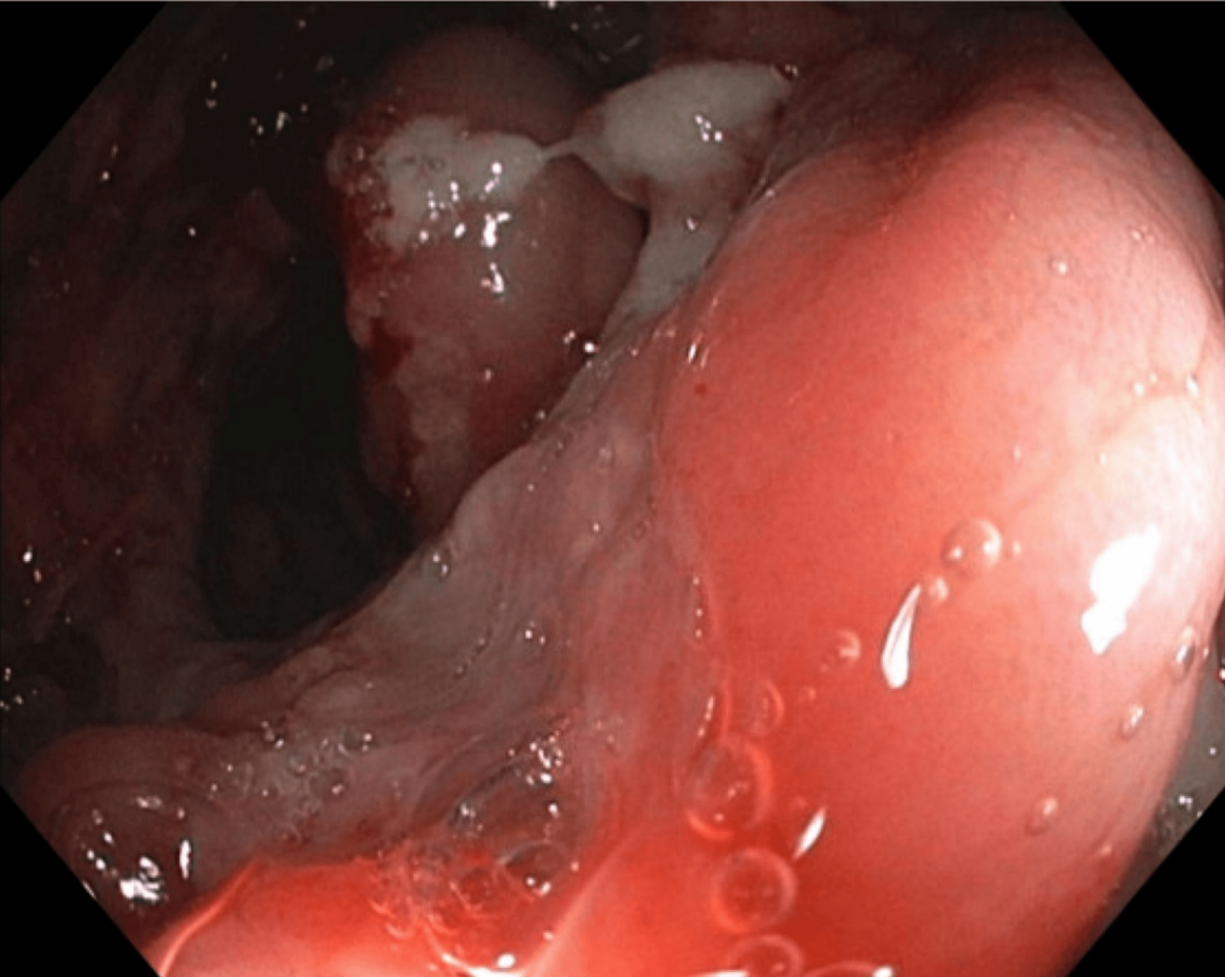
Cecum with severely erythematous, eroded, friable, inflamed, nodular, and pseudopolypoid mucosa

A diffuse area of severely erythematous, eroded, friable (with spontaneous bleeding), inflamed, nodular, and pseudopolypoid mucosa was found from the ascending colon (Figure 5) to the cecum (Figure 6).

Biopsies showed severe active chronic colitis with chronic granulomatous inflammation and ulceration. She was diagnosed with ileocolonic Crohn’s disease with scattered colonic involvement. She was started on combination therapy with methotrexate and infliximab; however, she developed headaches and severe nausea and vomiting with the methotrexate, and thus it was discontinued. Plans were to continue routine lab work every three to four months, including complete blood count (CBC), comprehensive metabolic panel (CMP), sedimentation rate, and CRP, to evaluate for disease activity and adverse effects of medications. She remained on infliximab for the following six months and has clinically responded well with adequate weight gain. She is mainly on a liquid diet; however, she has started to incorporate solids and has regained almost all the weight that she lost. Fecal calprotectin at this point was in the normal range.

## Discussion

This case highlights the multifactorial nature of FTT and the diagnostic challenge it can pose for clinicians. The patient had a complex presentation consisting of both organic and psychological symptoms, including gastrointestinal discomfort, chronic constipation, refusal to feed, and anxiety surrounding certain foods. The association between Crohn's disease and ARFID underscores the need for a holistic approach to treatment. ARFID can exacerbate the nutritional deficiencies and growth delays associated with Crohn's disease, further complicating the clinical picture [5,6]. In this pediatric patient, the parents noted that she was always a picky eater growing up, which could have been the insidious signs that an underlying disease process was taking place. Studies on the prevalence of ARFID have been estimated to range from 0.3% to 15.5%, while the incidence of IBD in children is estimated to be 7 per 10,000 children [7]. Crohn's disease often leads to symptoms, including gastrointestinal discomfort and weight loss, that can often mirror an eating disorder.

FTT in the pediatric population is typically diagnosed before the age of three, with the etiology often related to the development of the gastrointestinal tract. Manifestations of abnormal gastrointestinal development can vary widely, from issues with genetic malabsorption, chronic diarrhea, and abdominal discomfort. The workup for FTT includes ruling out organic causes, including, but not limited to, hematologic, thyroid, electrolyte, autoimmune, and radiographic imaging. This is in contrast to our patient, who largely had normal development until the age of 10. While the patient noted that symptoms of picky eating were prevalent growing up, it was not until she reached 10 years old that she began eating less of and even refusing her favorite foods. This contrast and the late development of FTT can be puzzling to the clinician and lead to the development of a theory that an acquired behavioral or psychiatric issue is the primary diagnosis.

It remains a challenge to identify if ARFID is the result of the underlying IBD or if the IBD led to the symptoms of ARFID. A study on 161 patients with IBD demonstrated a significant positive correlation with ARFID [4]. Additionally, psychological factors, including low self-esteem and depression, have been found to be associated with Crohn’s disease [8]. In those patients yet to be diagnosed with Crohn’s disease who manifest predominantly with symptoms such as depression and anxiety, abdominal discomfort is often attributed to a psychiatric disorder rather than an organic cause. Therefore, it is important for the clinician to consider a workup that includes both organic and inorganic causes.

This case emphasizes the importance of considering IBD in patients with ARFID, especially when accompanied by gastrointestinal symptoms such as blood in the stool or weight loss. The patient's response to treatment, including the improvement in her eating habits and weight gain, may highlight the effectiveness of a multidisciplinary approach to care.

## Conclusions

This case report illustrates the challenges and complexities of diagnosing and managing pediatric Crohn's disease with concurrent ARFID. It underscores the importance of a comprehensive and multidisciplinary approach to care. Early recognition and treatment of both Crohn's disease and ARFID are essential to prevent long-term complications and improve the quality of life for affected children. This case also highlights the need for further research to understand the relationship between IBD and eating disorders, which may lead to more effective treatment strategies.
